# Antioxidants in Sport Sarcopenia

**DOI:** 10.3390/nu12092869

**Published:** 2020-09-19

**Authors:** Maria Michela Cesare, Francesca Felice, Veronica Santini, Rossella Di Stefano

**Affiliations:** 1 Cardiovascular Research Laboratory, Department of Surgical, Medical and Molecular Pathology and Critical Care Medicine, University of Pisa, 56100 Pisa, Italy; maria.cesare@student.unisi.it (M.M.C.); rossella.distefano@unipi.it (R.D.S.); 2 Department of Life Sciences, University of Siena, via Aldo Moro 2, 53100 Siena, Italy; 3 S.D. of Sport Medicine, Pisa University Hospital, 56100 Pisa, Italy; veronic.santini@gmail.com; 4 Interdepartmental Research Center “Nutraceuticals and Food for Health”, University of Pisa, 56100 Pisa, Italy

**Keywords:** antioxidant, sport sarcopenia, muscle cells, nutritional supplements

## Abstract

The decline of skeletal muscle mass and strength that leads to sarcopenia is a pathology that might represent an emergency healthcare issue in future years. Decreased muscle mass is also a condition that mainly affects master athletes involved in endurance physical activities. Skeletal muscles respond to exercise by reshaping the biochemical, morphological, and physiological state of myofibrils. Adaptive responses involve the activation of intracellular signaling pathways and genetic reprogramming, causing alterations in contractile properties, metabolic status, and muscle mass. One of the mechanisms leading to sarcopenia is an increase in reactive oxygen and nitrogen species levels and a reduction in enzymatic antioxidant protection. The present review shows the recent experimental models of sarcopenia that explore molecular mechanisms. Furthermore, the clinical aspect of sport sarcopenia will be highlighted, and new strategies based on nutritional supplements, which may contribute to reducing indices of oxidative stress by reinforcing natural endogenous protection, will be suggested.

## 1. Introduction

Master athletes who engage in endurance physical activities, such as running and cycling, may suffer from muscle mass decrease over the years. This physiological condition may eventually evolve into pathological features, possibly resulting in sarcopenia, characterized by a reduction in strength and muscle mass.

In modern society, where life expectancy is continuously increasing, sarcopenia is most likely expected to occur; thus, representing an emergency healthcare issue of the future.

As first introduced by Rosenberg in 1988, to indicate “muscle loss” [1], sarcopenia is defined by structural, biochemical, molecular, and age-associated functional muscle changes. Over time, the definition has been revisited in order to take into account the fact that a loss of strength and muscle power is also associated with aging. Sarcopenia is one of the first implications of advanced age. It leads to weakness and an increased rate of falls, with obvious repercussions on the autonomy of movement and on the quality of life of those who are affected.

In 2019, Bauer J. et al. [2], refined the definition into primary and secondary sarcopenia, in order to improve the knowledge and recognition of the pathology.

Primary sarcopenia is a consequence of the aging process (e.g., reduced neurological function, altered muscle fiber distribution, and increased protein turnover). Secondary sarcopenia (or disease-related sarcopenia) predominantly focuses on loss of muscle mass, with no emphasis on muscle function.

Oxidative stress, characterized by an imbalance between reactive oxygen species (ROS) and reactive nitrogen species (RNS) levels, and the enzymatic antioxidant protection system, plays a key role in the pathophysiological mechanisms leading to sarcopenia

ROS/RNS have a double function in skeletal muscle. At low levels, they increase muscle force and adaptation to exercise, whereas at high levels, they lead to a decline of muscle performance. Exercise involves increased exposure to ROS and RNS. During exercise, skeletal muscle ROS is produced from both mitochondrial and non-mitochondrial sources, which include xanthine oxidases, NADPH oxidases, and phospholipase A2, whereas the parent RNS, nitric oxide (NO), comes from NO synthase (NOS) [3].

Sarcopenia can be counteracted with a change in eating habits as a way to overcome anabolic resistance of the muscle. Oral antioxidant supplementation may contribute to reducing the indices of oxidative stress, both in animal and human models, by reinforcing the natural endogenous defenses.

The present paper reviews the recent experimental models of sarcopenia, exploring the molecular mechanisms. Furthermore, the clinical aspect of sport sarcopenia will be highlighted, and new strategies based on nutritional supplements, will be suggested.

## 2. Skeletal Muscle and Sarcopenia

The skeletal muscle has a primary role in locomotion and in maintaining posture, representing 40% of total body mass. This tissue is responsible for normal physical activity, and is a key tissue involved in the regulation of metabolic homeostasis, by utilizing glucose and oxidizing fatty acids. The skeletal muscle responds, for example, to exercise or disuse, reshaping the biochemical, morphological, and physiological state of the individual myofibrils; thus, adapting to the new needs of the organism. Adaptive responses, which depend on the exercise stimulus, involve the activation of intracellular signaling pathways and, consequently, genetic reprogramming, which leads to alterations in contractile properties, muscle mass, and metabolic status [4]. For instance, resistance training increases muscle mass, strength, and fiber hypertrophy [5], whereas increased mitochondrial density and maximal oxygen uptake has been observed in endurance training [6].

The skeletal muscle is made up of different muscular fibers, which are multinucleated single cells, divided into four types of fiber in mammalian muscles, from the fastest (type IIB) to the slowest (type I). In particular, type I is a slow fiber, whereas types IIA, IIX, and IIB are the three fast fibers, each determined by the expression of one specific isoform of myosin heavy chain (MYH), a protein involved in the contractility phenomenon [7]. Muscle fibers differ, also, in their metabolic profile (from slow/oxidative to fast/glycolytic). Type I fibers contract slowly and use an oxidative metabolism, while type II fibers contract fast and are mainly glycolytic. Both IIA and IIB fibers are abundant in fast-twitch muscles and have high levels of glycolytic enzymes, leading to the classification reported above. However, in human muscles, fast fiber IIB is not detectable, even though the corresponding gene is present in the genome, and fibers identified as IIB (based on ATPase staining) are, in effect, IIX fibers based on MYH composition [7,8,9].

Lifestyle behaviors, such as smoking, an unhealthy diet, or lack of physical inactivity, as well as aged-related changes in cytokine levels and hormones, contribute to the onset of sarcopenia, and are, therefore, important risk factors. Sarcopenia is a multifactorial process characterized by inflammation, oxidative stress, motor neuron loss, and a change in endocrine function [10]. In particular, muscle protein metabolism is a dynamic process characterized by the balance between the synthesis and breakdown of muscle proteins. As reported by Fry et al. [11], a disturbance of the equilibrium between muscle protein synthesis and breakdown can lead to the loss of muscle mass, and a perturbation of muscle protein turnover with aging can play a role in the development of sarcopenia. Sarcopenic muscle is primarily due to a loss of muscle fibers characterized by a preferential atrophy of type II fibers. Furthermore, a conversion of fast type II muscle fibers into slow type I fibers has been described, with a consequent loss in muscle power and decline in protein synthesis [12]. Proteins are a key component of any diet and there should be a sufficient amount to assure muscle mass. Protein ingestion, and the subsequent hyperaminoacidemia, stimulates muscle protein synthesis (MPS) rate and inhibits muscle protein breakdown rates, likely via the insulin pathway. In the past years, athletes were advised to follow the recommended dietary allowance (RDA) regarding protein (i.e., from 0.8 to 1.0 g/kg/d for children, adolescents, and adults) [13]. Recent studies have shown that protein intake must be proportional to the exercise type, intensity, and the quality of the protein ingested. In endurance sports, the athlete needs a higher value of RDA protein (1.5 to 2.0 g/kg/d) to maintain the nitrogen balance and avoid muscle catabolism [14]. For people who do general fitness programs, it is recommended they consume 0.8–1.0 g/kg/d of protein. For athletes who practice moderate activities, the recommendation is to consume between 1 and 1.5 g/kg/d protein; for athletes who do intense levels of activity, the recommended protein intake is 1.5–2 g/kg/d [15].

Balanced protein intake prevents the risk of incurring sarcopenia. The position statement (for protein) from the International Society of Sports Nutrition (ISSN) coordinates with the Acceptable Macronutrient Distribution Range, and is published by the Institute of Medicine—it states that, to preserve muscle mass, a daily protein intake between 1.4–2.0 g/kg/d is necessary for most training individuals [16].

Recently, Traylor et al. [17] reviewed the optimal dietary protein intake to stimulate MPS, particularly in older individuals, proposing that ≥ 1.2 g/kg/d should be consumed, with an emphasis on the intake of the amino acid leucine, which plays a central role in stimulating skeletal muscle anabolism. The reduced sensitivity of the muscle in response to amino acids and protein in older persons may be the cause of at least one of the following factors: reduced physical activity levels, impaired protein digestion and amino acid absorption, increased splanchnic amino acid retention leading to reduced aminoacidemia, impaired muscle perfusion reducing delivery of amino acids to the muscle, reduced uptake of amino acids by the muscle, and impaired intracellular anabolic signaling [18]. In sarcopenic muscle a reduction in the number of myofibers and hypotrophic myofibers, as well as infiltration into adipose and, at later stages, fibrotic tissue, has also been observed. A decrease in the number of satellite cells (SCs), the adult stem cells of skeletal muscles located between the sarcolemma and the basal lamina (essential for the maintenance of muscle mass), has also been observed [19].

The loss of strength is not always directly related to the loss of muscle mass. Factors related to the quality of muscle may contribute to a decline in contractile capacity; however, the interaction of these various features has not been assessed fully. The fattening of the muscles was previously shown to be correlated with muscle weakness and functional deterioration [20]. In particular, Visser et al. [20] observed an association between lower muscle mass, greater fat infiltration into the muscle, and an increased risk of mobility loss in older men and women. In fact, when myosteatosis, an increased deposition of intramuscular fat with replacement of muscle tissue, occurs after deterioration of muscle quality, an adverse impact on metabolism and peak force generation arises. Myosteatosis triggers a transition of muscle fibers from type II to type I, reducing the power and contractile capacity of the muscles [21]. Finally, Barbat-Artigas et al. [22] observed an inverse relationship between muscle mass and quality, and concluded that sarcopenic individuals have better muscle quality than nonsarcopenic individuals, and that high muscle quality may compensate for low appendicular skeletal muscle mass index, with respect to functional damage.

## 3. In Vitro Sarcopenia Induction

Numerous studies have shown that glucocorticoids decrease muscle size, largely due to accelerated muscle protein breakdown, a mechanism linked to an increased catabolism of muscle proteins by the ubiquitin-proteasome pathway [23,24]. It has been observed that different pathologic conditions can stimulate endogenous glucocorticoid production, such as inflammation, burn injury, diabetes, sepsis, metabolic acidosis, and high angiotensin II levels [25,26]. In a recent study, Klein [27] reported the effect of both endogenous and exogenous glucocorticoids on bone and muscle, demonstrating that the supraphysiologic loads of glucocorticoids are similar, regardless of whether they enter the body as medication, or are produced by the body in response to stimuli, such as inflammation. In particular, protein degradation occurs by the covalent attachment of the protein ubiquitin, by ubiquitin E3 ligases, namely muscle atrophy F-box (MAFbx, also called atrogine-1) and muscle ring-finger 1 (MuRF1). MAFbx and MuRF1 are two muscle-specific ubiquitin ligases that have been linked to muscle atrophy when upregulated [28,29,30]. In addition to regulating protein breakdown, both MuRF1 and MAFbx may also contribute to a decrease in protein synthesis in response to glucocorticoid administration [31]. Thus, to mimic in vitro sarcopenia, treatment with dexamethasone, a synthetic glucocorticoid, is used. The classical mechanism of action for glucocorticoids occurs through the glucocorticoid receptor (GR), a steroid hormone receptor that regulates gene expression through direct and indirect interactions with chromatin. As reported by Shimizu et al. [32], fast-twitch muscles contain a greater GR density than slow-twitch muscles, suggesting that slow-twitch fibers may be spared in glucocorticoid toxicity. Moreover, the authors observed that mammalian target of the rapamycin (mTOR) activation inhibits GR transcription function; thus, counteracts the catabolic processes provoked by glucocorticoids. The glucocorticoids that bind to their receptors can activate genomic and non-genomic pathways.

**Figure 1:**
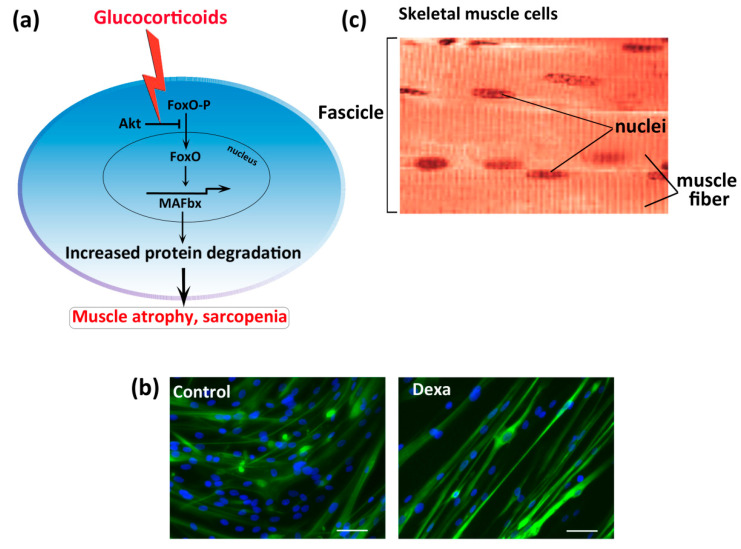
Sarcopenia induced by glucocorticoid-treated skeletal muscle cells in vitro. (**a**) Protein kinase B (Akt) causes phosphorylation and nuclear exclusion of Forkhead box protein O (FoxO) family, which suppresses atrogene (muscle atrophy F-box, MAFbx) expression and proteolysis. Dexamethasone prevents Akt-mediated phosphorylation of FoxO (FoxO-P), inducing its transfer to the nucleus and the consequent MAFbx upregulation and protein degradation. (**b**) The skeletal muscle structure. (**c**) Representative images of human myotube after dexamethasone (Dexa) treatment (50 µM for 48 h), showing fluorescence double labeling, using an anti-myosin heavy chain-2 antibody (green), and Hoechst (nuclei in blue). Scale bar = 100 µm.

The non-genomic mechanism involves GR-independent intercalation into the cytoplasmic membrane or interactions of the GR with kinases or their regulatory molecules [33,34]. Numerous studies have shown the non-genomic mechanisms of action induced by dexamethasone in myotube atrophy. Clarke et al. [35] observed that dexamethasone stimulates the degradation of MYH protein through the action of MuRF1, demonstrating that the inhibition of a single E3 ligase, MuRF1, is adequate to maintain this important sarcomeric protein. Moreover, the involvement of the phosphoinositide 3-kinase/protein kinase B signaling pathway (PI3K/Akt) has been demonstrated in myotube atrophy induced by glucocorticoids. In particular, FoxO1 and FoxO3 have been implicated in muscle wasting [36]. The Akt-mediated inhibition of the FoxO family of transcription factors is reduced by dexamethasone, which prevents Akt phosphorylation and leads to activation of FoxO transcription factors and MuRF1 and MAFbx upregulation [36,37,38] (Figure 1). Due to the critical role of FoxO transcription factors in the development of muscle atrophy, therapeutic approaches to inhibit FoxO phosphorylation are of clinical interest to counteract muscle wasting. However, the above mechanism has been demonstrated in animals, but its effect in humans has not yet been determined.

## 4. Diagnosis of Sarcopenia

The difficulty of quantifying sarcopenia with accessible and practical methods is well known. Diagnosis of sarcopenia relies on the combined measurement of muscle mass: the gold standard is whole-body multislice magnetic resonance imaging (dual energy X-ray absorptiometry, DEXA). An alternative method to DEXA is a less valid predictive technique, such as bioelectrical impedance analysis (BIA). Both DEXA and BIA methods are combined with other measurement methods, such as muscle strength, assessed by functional tests, such as grip strength and physical performance measured by gait speed, low physical performance battery (SPPB), Timed Up and Go test, or 400 m walk [39]. However, many research groups are attempting to define better strategies [40,41,42]. Lee CR et al. [43] have developed two anthropometric prediction models in vivo by using state-of-the-art body-composition methods; these proved to be useful in clinical evaluations of skeletal muscle mass in adults. One of the formulas used in these anthropometric models is [43]:

SM (kg) = Ht (0.00744 CAG2 + 0.00088 CTG2 + 0.00441 CCG2) + 2.4 sex 0.048 age + race + 7.8(1)

where SM = skeletal muscle mass, R2 = 0.91, *p* < 0.0001, and SEE = 2.2 kg; sex = 1 for male and 0 for female, race = 2.0 for Asian, 1.1 for African American, and 0 for white or Hispanic. SM is whole-body SM (in g), Ht is height (in cm), CAG is corrected arm girth (in cm), CTG is corrected thigh girth (in cm), and CCG is corrected calf girth (in cm).

The Strength, Assistance with walking, Rise from a chair, Climb stairs and Falls SARC-F questionnaire, consisting of five questions, is recommended for the screening of sarcopenic subjects. It is a rapid screening tool that should be utilized with a formal diagnosis made by measuring grip strength or chair stand, together with DXA estimation of appendicular muscle mass [2,44]. Measuring the circumference of the calf can also improve the specificity of SARC-F. An alternative screening test is the Ishii test (age, grip strength, and calf circumference) [45,46].

However, as observed by Rolland et al., “All definitions of sarcopenia are arbitrary and open to criticism. An operational definition needs to differentiate those with sarcopenia from those not affected, needs to define standards and be applicable across populations” [47].

## 5. Recommendations of Physical Activity and Classification of Sport Disciplines

Physical activity has many benefits, reduces cardiovascular risk, and improves the quality and expectancy of life. The current European [48] and American [49] recommendations concerning physical activity indicate 150 min per week of moderate physical exercise (30 min/day, 5 days/week) or 75 min/week of physical exercise of vigorous intensity (15 min/day, 5 days/week). Practicing physical exercise is recommended at a frequency of at least three to five sessions per week; the best results, in terms of health benefits, occur with a daily workout and a combination of exercise of both moderate and vigorous intensity, performed in sessions with a duration of at least 10 min [50].

Competitive (and some recreational) athletes exercise in excess of these recommendations and regularly train more than 20 h of intense exercise (15 MET or metabolic equivalents) per week. According to the scientific statement of the American Heart Association/American College of Cardiology (AHA/ACC) [51], a competitive athlete is defined as “one who participates in an organized team or individual sport that requires regular competition against others as a central component, places a high premium on excellence and achievement, and requires some form of systematic and usually intense training” [52]. From a quantitative point of view, an athlete may be considered “elite” if competing regularly at the regional, national, or international level, and exercising for at least 6 h/week. Additionally, it is important to remark that some people who do sports only for recreational purposes can reach training levels comparable to those of professional athletes [53].

Physical exercise can be classified into two large groups: dynamic and static. Dynamic exercise is defined as isotonic because muscle length and joint movement with rhythmic contractions develop a relatively small intramuscular force. Isometric (static) exercise creates a relatively large intramuscular force with little or no change in muscle length or joint movement. Most sport disciplines involve both static and dynamic components, so it is necessary to consider other variabilities. Important aspects are the type of metabolism activated (aerobic and anaerobic), intensity (low, moderate, high), duration of activity, and both acute and chronic cardiac adaptation [54].

Endurance sports are characterized by aerobic and prolonged exercise (Table 1). In an acute phase of endurance training, there is an increase in cardiac output, maximum oxygen consumption, and peripheral vasodilatation with a reduction in the oxygen demand of tissues [55]. Strength or isometric exercise, such as weightlifting, is an anaerobic exercise that implies the enhancement of strength, anaerobic work, and dimension of skeletal muscles. The increment in oxygen consumption and the cardiac output are not predominant during this type of exercise; however, there is a prevalent increase of blood pressure, heart rate, and peripheral vascular resistance.

## 6. Sarcopenia in Ultra-Endurance Athletes

### 6.1. Effects of Endurance Training on Body Muscle Mass

Ultra-endurance performance is described as an event exceeding six hours in duration. During this kind of training, it is necessary to have a long-term preparation, sufficient nutrition, and balance between multiple stressful factors, such as thermal stress, muscle damage, central fatigue, and limited endogenous muscle glycogen content [56]. The main acute effects of ultra-endurance exercise are increased concentrations of interleukin 6 (IL-6) and white blood cells (WBCs), which are proportional to type, intensity, and time of exercise [57]. Anthropometric aspects (i.e., body mass, body fat, fat-free mass), training characteristics (i.e., intensity, frequency, and duration of training), and physiological variables (i.e., maximum oxygen uptake) are important predictor elements for competition performance. Over the last decades, the interest in age-related change on endurance performances has increased [58,59]. In recent years, the number of master athletes (over 40 years old) has risen, especially for endurance sports, such as swimming, cycling, and running. Competitors prefer multi-sport disciplines (i.e., duathlon, triathlon, pentathlon, etc.) and longer running distances, such as half-marathons, marathon, and ultra-marathons [60].

It has been demonstrated that endurance performance decreases with increasing age. Up to the age of 30–35 years, endurance athletes achieve the best results, after which there is a moderate decline until the age of 50–60 years [61,62].

Thus, if, on the one hand, extreme and prolonged exercise results in energy deficits expressed by anthropometric change in body mass (muscle loss), body fat, and fat-free mass, then, on the other hand, lifelong physical activity can produce loss in skeletal muscle structure and function [63].

Ultra-endurance competitions are a good opportunity to study anthropometric changes in long lasting endurance performances. Ultra-endurance training, for hours or even days or weeks (especially without defined breaks), produces a decrease in body mass as well as skeletal muscle mass. Knechtle et al. [64] demonstrated that competitions with defined breaks and non-stop performances lead to different body adaptations. In particular, they investigated the change in body composition in ultra-endurance runners during the Isarrun 2006 in Germany. The competition was planned in five stages (five consecutive days) in which the athletes had to run 338 km. There were fifty-two males and eight females at the start; fifty male and seven female runners finished the competition. The study focused on twenty-one male Caucasian runners (age 41.5 ± 6.9 years old). In contrast with another case report that described the decrease in body mass in athletes of non-stop ultra-endurance races (between 1.75 kg in a multi-day run over 1000 km) [65], after the Isarrun 2006 competition, ultra-endurance runners showed stable values of body mass, but a significant decrease in skeletal muscle mass. The method used to evaluate ultra-endurance runners during the Isarrun 2006 was the formula (1).

The calculated skeletal muscle mass during the first stage showed the most apparent decline. The conclusion of the research article was that a multi-stage ultra-endurance run (over 338 km within five days) leads to no changes in body mass or body fat mass, but generates a statistically significant decrease in skeletal muscle mass.

Despite the evidences about acute effects of ultra-endurance exercise (as in races, competitions), the study of body composition variation during training and preparation for an ultramarathon race can produce significant information [66]. The day-by-day evaluation of body composition during training and preparation of an ultra-marathon race proved a significant decrease in body mass, body fat, and fat-free mass. The case report shows that the training plan of master athletes must follow a progressive pattern with regard to exercise intensity and training volume, which increase over time [67,68].

### 6.2. Effects of Aging on Athletes and Body Muscle Mass

A study concerning the effects of aging on athletes, mostly in male subjects [62,69,70], suggests that the realization of an optimal training status to prevent fiber muscle damage does not protect from sarcopenia [59]. Elderly endurance-trained athletes have to know that prolonged and extreme exercise causes a decline in skeletal mass and that age-associated atrophy, fatigability, and weakness can be slowed down, but not arrested. Progressive loss of muscle mass and strength, and performance in speed and force events in master class athletes (who continue to compete for their entire adult lives) occurs after the age of 30 [71]. In marathon runners and weightlifters of master class athletes, the decrease occurs later after, at about 40 years of age, with peak performance rates of about 50% for 80 years of age [72]. Studies on the general population have demonstrated that the loss in skeletal muscle mass is more pronounced in men aged 60, as compared to women of the same age, with sarcopenia present in ~53% of men compared to ~47% of women [73]. In people over 80, the prevalence of sarcopenia was ~31% in women and ~53% in men [74]. Considering athletes, in 2016, Knechtle et al. [75] investigated 65,584 freestyle master swimmers aged 25–89 years, competing in the FINA World Masters Championships between 1986 and 2014. The authors evaluated the trends in participation, performance, and sex difference. When the interaction effects between sex and distance were considered, the authors observed that women were not slower compared to men in the 90–94 year-old master group, hypothesizing a role of the anthropometric differences due to the diverse fat-free mass between women, with respect to age. Fat-free mass, in fact, remained stable up to 60 years of age in men, and was lower at 75 years of age compared with younger men. In women, it was lower from the age of 60 [76].

### 6.3. Exercise in the Prevention of Sarcopenia

Physical exercise is just one of the beneficial factors of an adequate lifestyle, and can reduce the risk of various pathologies. The negative effects caused by free radicals are alleviated by regular exercise. As stated above, intensive exercise promotes increased ROS production, contributing to the development of acute muscle fatigue.

The role of the different types of exercises in sarcopenia has been shown [77]: aerobic exercise provides a partial solution to sarcopenia since it ameliorates mitochondria-derived problems, improving oxidative capacity, whereas resistance exercise fortifies muscle mass and function, increasing muscle mass, muscular strength, and functional abilities [78,79]. Endurance exercise has been recommended and reported to improve muscle function [80,81].

**Figure 2:**
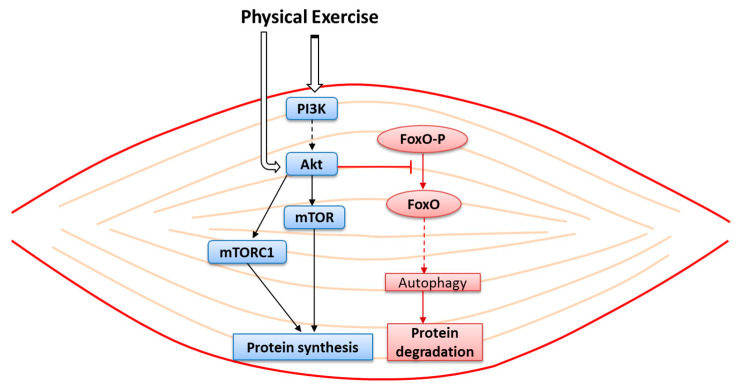
A schema of the intracellular pathway activated by physical exercise in skeletal muscle cells, showing protein synthesis (blue) and protein degradation pathway (pink). The solid line represents direct reaction; the broken line represents the intermediate pathway and products of reaction. Phosphoinositide 3-kinase (PI3), protein kinase B (Akt), mammalian target of the rapamycin (mTOR), mTOR complex 1 (mTORC1), Forkhead box protein O (FoxO) and phosphorylated forkhead box protein O (FoxO-P).

Exercise is important in the prevention of sarcopenia because it is involved in the activation of the mammalian target of the rapamycin (mTOR) signaling pathway in skeletal muscle [82]. The mTOR is a central cell growth regulating kinase, acting in a structural and functional complex, namely mTOR complex 1 (mTORC1), the architecture of which has recently been described [83]. The mTOR signaling pathway in skeletal muscle is associated with an increased muscle protein synthesis during the early recovery phase following a bout of resistance exercise (Figure 2). Resistance exercise, in particular, activates mTORC1 and muscle protein synthesis [84]. Moreover, it has been demonstrated that resistance exercise is a powerful anabolic muscle stimulus, which increases myofibrillar muscle protein synthesis. After a single period of resistance exercise, muscle protein synthesis increases within 2–3 h; it remains higher up to 24 h in trained individuals and up to 48 h in untrained subjects [85].

It important to note that other signaling pathways are activated or inactivated during oxidative stress and molecular inflammation in aged skeletal muscle, leading to sarcopenia. Meng and Yu [86] reviewed the major signaling pathways involved in sarcopenia and inflammation. For instance, the aging-related redox-sensitive transcription factor NF-*κ*B (or nuclear factor kappa-light-chain-enhancer of activated B cells) is activated by ROS either directly or indirectly. The activated NF-κB regulates, at the nuclear level, the expression of myogenic differentiation 1 (MyoD) and other molecules, such as MuRF1, inducing muscle atrophy [87]. Different studies have provided robust evidence that NF-κB can serve as an important molecular target for the prevention of skeletal muscle loss [87]. Moreover, the Transforming Growth Factor-Beta (TGF-β) signaling pathway has an important role in sarcopenia, since muscle regeneration is modulated by members of the TGF-β superfamily, one of which is myostatin, a myokine and secreted growth differentiation factor that regulates muscle growth, upregulating the ubiquitin ligases atrogin1 and MuRF1 via FoxO transcription factors [88].

Consistent improvements in lower body muscle strength are evident after training and exercise combined with diet [89]. In order to balance the energy deficit and following anthropometric changes (skeletal muscle mass reduction), the essential role of optimal nutritional strategies has also been demonstrated [90]. It is known that dietary supplementation helps athletes in the preparatory phase of a competition [91].

Future studies are needed to clarify the volume and intensity of exercise to effectively prevent the progression of sarcopenia.

## 7. The Use of Antioxidants in the Prevention of Sarcopenia

### 7.1. Exercise and ROS Production: How to Prevent Oxidative Damage

As first reported by Davies et al. in 1982 [92], numerous studies have subsequently reported that strenuous exercise and endurance training causes ROS and RNS accumulation in skeletal muscle, which promote oxidative stress [93,94,95]. Oxidative stress is the result of an imbalance between the physiological production of free radicals and the cells ability to scavenge them. ROS, such as superoxide anions, hydrogen peroxide, and hydroxyl radicals, are constantly produced by muscle cells, both in resting conditions and during contraction, controlling force production [96]. The consequent pro-oxidant state causes the alteration of the mitochondrial DNA and some anomalies in the electron transport system, leading to a reduction in the absorption of calcium by the sarcoplasmic reticulum and irreversible damage to the cell and its consequent death [97].

A moderate increase in the levels of ROS, which can be observed from light to moderate exercise, causes an increase in the development of muscle strength up to a maximum peak; however, a further increase induces a drastic decline in strength [98,99].

**Figure 3:**
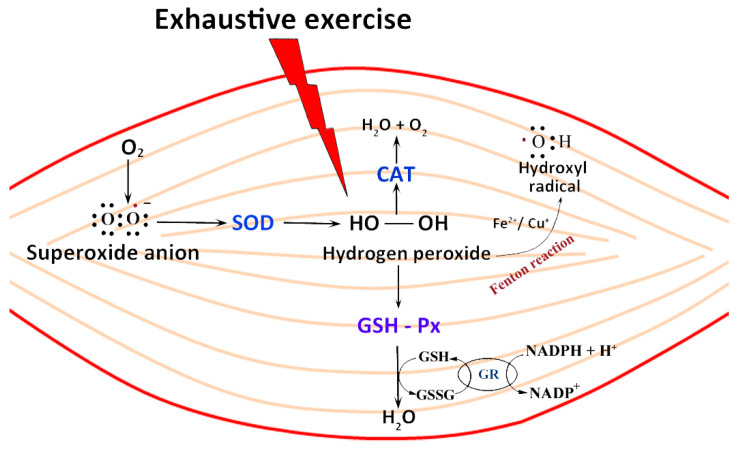
Antioxidant enzyme activity to counteract reactive oxygen species (ROS) production and accumulation in skeletal muscle cells after exercise. Exhaustive exercise induces an increased production of reactive oxygen species: superoxide anion (O^2-^), hydrogen peroxide (H_2_O_2_) and hydroxyl radical (OH.) according to the Fenton reaction. Catalase (CAT), glutathione transferase (GSH), glutathione peroxidase (Px), and superoxide dismutase (SOD), work to maintain a state of oxidative balance, producing water and oxygen. Glutathione disulfide (GSSG), Glutathione reductase (GR), nicotinamide adenine dinucleotide phosphate hydrogen (NADPH) and nicotinamide adenine dinucleotide phosphate (NADP).

ROS are produced in muscle cells by various fonts in different cell compartments and through different pathways. During exercise, the mitochondrial electron transport chain is one of the main sites of ROS production [100]. Due to high oxygen consumption by increased mitochondrial activity, the transfer of a single electron to molecular oxygen gives rise to a monovalent reduction of oxygen, which leads to the formation of superoxide ions. The enzymatic process can also promote superoxide production through NADPH oxidase enzymes or the xanthine/xanthine oxidase system [101]. Moreover, in the presence of transition metal ions (e.g., Fe^2+\3+^, Cu^+\2+^), hydrogen peroxide (H_2_O_2_) produces the highly reactive hydroxyl radical (OH.) and hydroxyl ion (OH-), according to the Fenton reaction [102] (Figure 3).

To prevent oxidative cell damage, a well-organized system of antioxidants act in a synchronized fashion. Cells, including muscle fibers, contain a network of antioxidant defense mechanisms to reduce the potential of oxidative damage during periods of increased ROS production. The enzymatic and non-enzymatic (i.e., vitamin A, vitamin C, vitamin E, β-Carotene) systems regulate the homeostasis redox status of muscle cells. Catalase (CAT), glutathione transferase (GSH), glutathione peroxidase (GPx), and superoxide dismutase (SOD), are some of the components of the enzyme elimination system that are significantly depressed in elderly muscle. Generally, antioxidant enzymes (e.g., CAT, SOD, GPx, or GSH) work to maintain a state of oxidative balance, converting ROS into more stable molecules, such as water and molecular oxygen (Figure 3). Most studies investigating the adaptive responses to exercise-induced ROS/RNS generation have shown that both acute and regular exercise [103] induce increased activities of antioxidant defense enzymes in skeletal muscle, in particular SOD [104]. Existing in the cytoplasm of the eukaryotic cells and in the mitochondria (as Cu/ZnSOD and MnSOD, respectively), SOD is involved in the dismutation of superoxide anion in molecular oxygen and hydrogen peroxide [105], which is converted into water by catalase or GPx. In particular, an increase in SOD protein content has been observed during repeated bouts of aerobic exercise in mice muscle [103].

In the reaction catalyzed by GPx, glutathione is oxidized to glutathione disulfate, which can be converted to glutathione by glutathione reductase in an “NADPH-consuming” process [106]. Catalase (CAT) is a high molecular weight tetrameric enzyme containing porphyrin in the active site. It is one of the fundamental antioxidant enzymes that mitigates oxidative stress to a significant extent by destroying cellular hydrogen peroxide to produce water and oxygen [107] (Figure 3).

Antioxidants play important roles in regulating ROS levels through direct free radical scavenging mechanisms, through regulation of ROS/RNS-producing enzymes, and/or via adaptive electrophilic-like mechanisms. However, the relationship between free radical generation, antioxidant enzymes, and exercise in skeletal muscle remains controversial, due to the differences in exercise intensity, mode, duration of training program, and muscle fiber type. In fact, each muscle fiber type has distinct oxidative potential and metabolic characteristics as well as antioxidant defense capacity [108]. As reported by Gonchar [109], in a study carried out on rats, after endurance swimming training, a significant decreases in GPx, glutathione reductase activities and GSH content in both fast- and slow-twitch muscles was observed, confirming that antioxidant enzyme response to chronic exercise is highly muscle fiber specific.

To reinforce the natural endogenous protection, nutritional supplements may represent a good strategy, contributing to reducing the indices of oxidative stress. Nutritional antioxidants act in different mechanisms and compartments: (1) neutralize free radicals; (2) repair oxidized membranes; (3) decrease ROS production; and (4) neutralize ROS via lipid metabolism, short-chain free fatty acids, and cholesteryl esters [110]. Moreover, dietary antioxidant vitamins (such as C, E, and carotenoids) are auspicious candidates for the prevention of age-related loss of mass and function.

### 7.2. Exogenous Antioxidant

Exogenous antioxidants have generated growing interest in preventing oxidative stress, in decreasing muscle pain and physical stress, and in improving sport performance. It is important to note that higher dosages of antioxidants may not necessarily be beneficial for athletes training for and competing in different sporting events, but can also elicit detrimental effects by interfering with performance-enhancing and health-promoting training adaptations [111].

Exogenous antioxidants act in addition to the endogenous ones. Tocopherols or vitamin E, L-ascorbic acid or vitamin C, carotenoids, ubiquinone, and polyphenols are the most well-known exogenous antioxidants [112]. Although natural compounds with antioxidant activity are numerous, we reported the most known nutritional antioxidants, mainly vitamin C, vitamin E, carotenoids, flavonoids, and polyphenols [113,114,115,116], whose functions on muscles have been more extensively studied. According to the hydrophobicity of the administered molecule, the supplementation of different antioxidants will provide different effects on oxidation. For example, fat-soluble antioxidants, such as vitamin E, are mostly effective in inhibiting lipoprotein peroxidation, while water-soluble antioxidants, such as vitamin C, are better able to protect the aqueous phase. On the other hand, these antioxidants also act cooperatively and sometimes even synergistically.

Vitamin C, an electron donor (reducing agent), whose antioxidant function derives from its ability to reduce oxidized species or oxidant radicals [117], is an indispensable nutrient, which plays a critical role in multiple hydroxylation reactions, maintaining redox homeostasis in organelles, such as mitochondria and the endoplasmic reticulum [118], and is one of the most important antioxidants in human plasma [117]. The effect of vitamin C supplements on antioxidant defense in human skeletal muscle during training has been widely studied. Khassaf et al. [104] observed that antioxidant defense mechanisms of vitamin C were more specifically on SOD and CAT activity; thus, improving skeletal muscle antioxidant defense. Very recently, Welch et al. [119] studied the effect of antioxidants, particularly vitamin C, on loss of skeletal muscle mass and power during the aging process. The authors investigated the associations between an extensive range of sarcopenic indices, including DXA-measured fat-free mass, grip strength, and leg explosive power, and a range of antioxidant vitamins (A, C, E, and carotenoids), observing a significant improvement of sarcopenic indices of skeletal muscle after antioxidant intake.

Unlike most mammals, and other animals, humans cannot synthesize vitamin C because they lack the enzyme l-gulonolactone oxidase in the biosynthetic process. Therefore, it must be obtained by dietary intake, particularly from fruits and vegetables. The principal sources of vitamin C are kiwifruit, broccoli, kale, strawberries, tomatoes, and sweet red pepper [120]. In our opinion, athletes should consume more vitamin C-rich food, such as broccoli (60 mg / 100 g vitamin C), blackcurrants (130 mg/100 g), fortified breakfast cereals (up to 134 mg/100 g), and oranges (37–52 mg/100 g), adding supplements to their diet only if necessary. Chronic supplementation (i.e., more than 2 weeks) with vitamins C and E, either individually or in combination, during exercise training, have been the most widely studied supplementation. Neubauer et al. [121] discussed the findings of a number of key studies and their implications for defining guidelines for the intake of vitamin C and E in athletes.

The most common isoform of vitamin E in the human body is α-tocopherol, although vitamin E includes eight fat-soluble isoforms. Rich sources of α-tocopherol are sunflower seeds, almonds, and hazelnuts, as well as many vegetable oils (e.g., olive oil and canola oil) and vegetables (e.g., tomato, spinach, and asparagus). The number of methyl groups on the chroman ring is responsible for the antioxidative effects of vitamin E isomers. In particular, three methyl groups make up α-tocopherol, while one methyl group in the chroman ring organizes δ-tocopherol. The order of α > β > γ > δ indicates the strength of antioxidative activity between vitamins E isomers, and α-tocopherol is stronger than tocotrienol [122].

Peroxyl radical are intercepted by α-tocopherol, preventing lipid peroxidation and the negative effects of free radicals in membranes and plasma lipoproteins [123]. Recently, Miyazawa et al. [124] reviewed the role of vitamin E in redox interaction, reporting its relationship with ROS and the antioxidative mechanism of action. However, the antioxidant effects exerted by vitamin E are complicated; furthermore, the mechanisms are not yet well understood.

Rokitzki et al. (1994) [125] reported that administration of combined vitamins C and E for 1 month prior to a marathon race decreased the indices of muscle damage after the race. However, consuming high doses of single antioxidants (such as vitamins C and E) may inhibit the signaling pathways normally triggered by the oxidative stress of exercise during training [91].

Recent human studies have investigated the interrelation of antioxidant supplementation and exercise training, using a more sophisticated design, methodologies, and techniques, and focusing not only on performance, but also on the health aspects of endurance training.

Carotenoids, a terpenoid-based compound, are organic pigments present in fruits and vegetables, such as pumpkins, carrots, corn, and tomatoes, representing important dietary antioxidants. Few carotenoids (about a dozen) are known to be healthy components for the human diet. Among these, beta-carotene and several xanthophylls, such as lutein, without or with oxygen in the molecule, respectively, are included. Recently, Bohn et al. [126] reviewed the benefits of carotenoids in humans. They evaluated the role of carotenoids in human chronic diseases characterized by oxidative stress, and reported that the beneficial health effects of carotenoids shown in small-scale studies and in subjects suffering from chronic oxidative stress are not evident in large-scale trials with carotenoid supplements. Finally, as recently evidenced by Sandmann et al. [127], carotenoids can play a role as direct antioxidants, by quenching singlet oxygen and peroxide radicals.

Polyphenols are a group of water-soluble, plant-derived substances, characterized by the presence of more than one phenolic group [115]. These molecules are divided into two sub-categories: flavonoids and phenolic acids. There are at least six subclasses of flavonoids, often referred to together, based on distinct differences in chemical structure. Flavonols are present in a wide variety of fruits and vegetables. Quercetin and kaempferol are the most common flavonols in food [128].

Polyphenols have potent free radical scavenging abilities. However, the in vivo relevance of these mechanisms is debatable due to their generally poor oral bioavailability with transient low accumulation [129], relatively poor uptake into peripheral tissues, such as skeletal muscle [130], and poor competition for ROS/RNS with endogenous antioxidants. Ingested polyphenols undergo extensive metabolism and modification in the liver and intestines. Resveratrol, a natural polyphenol found in grapes, peanuts, and berries, has shown a protective effect against oxidative stress in skeletal muscle through the expression of antioxidant enzymes [131]. Moreover, evidence in rodent skeletal muscle has shown improvements in oxidative stress and induction of endogenous antioxidant enzymes after resveratrol supplementation when combined with exercise [132,133,134].

Recently, Myburgh K.H. [135] reviewed the benefits of polyphenol supplementation for exercise performance, paying attention to the details of study design, subject population, supplementation regimen, and testing protocol aspects (and not only the outcomes of the various studies). The author affirmed that “although it is clear that polyphenol supplementation in a variety of forms and doses is able to increase the capacity to quench free radicals, at least in the circulation, it is not yet clear whether it holds beneficial effects for athletes” [135].

Finally, even after many years of research, it is not possible to draw clear conclusions, as a number of studies have not been able to clearly demonstrate excessive damaging effects of exercise with or without antioxidant supplementation.

## 8. Conclusions

With the diffusion of amateur and professional sports activities among citizens of developed countries, an increase in the emergence of sarcopenia is expected in the future. Sarcopenia involves the loss of muscle mass and strength during the aging process, with consequences on one’s balance, gait, and overall ability to perform daily living tasks.

Oxidative damage has been proposed as one of the major contributors of skeletal muscle decline. While physical activity itself can lead to sarcopenia, physical exercise combined with a regular intake of exogenous antioxidants can prevent the occurrence of the pathology. In fact, the identification of free radicals as promoters of sarcopenia in older master athletes could imply that their inhibition might limit the detrimental modifications to skeletal muscle.

Interventions aimed at enhancing the endogenous antioxidant defenses (e.g., dietary antioxidant supplementation) may gain special interest.

Despite the clinical relevance of sarcopenia, and the great interest for antioxidant supplementation, the effect of antioxidant supplementation on muscle performance is still largely controversial.

Thus, further studies are needed to define common guidelines for antioxidant supplementation to prevent sport sarcopenia, testing the efficiency of the treatment, and increasing the age sample of the studied subjects.

## Figures and Tables

**Table 1:** Endurance sports.

Sports
canoeing
cross-country
cycling
mid and long distance swimming
mid and long distance running, long distance skating
biathlon
triathlon
pentathlon
rowing
jogging
long distance walking
speed walking
style dancing
